# Osmodehydrofreezing: An Integrated Process for Food Preservation during Frozen Storage

**DOI:** 10.3390/foods9081042

**Published:** 2020-08-02

**Authors:** Maria C. Giannakourou, Efimia K. Dermesonlouoglou, Petros S. Taoukis

**Affiliations:** 1 Department of Food Science and Technology, University of West Attica, 12243 Egaleo, Greece; 2 National Technical University of Athens, School of Chemical Engineering, Laboratory of Food Chemistry and Technology, 15780 Athens, Greece; efider@chemeng.ntua.gr (E.K.D.); taoukis@chemeng.ntua.gr (P.S.T.)

**Keywords:** osmotic dehydration, freezing, kinetic study, frozen food quality, quality loss kinetics, alternative osmotic agents, cold chain

## Abstract

Osmodehydrofreezing (ODF), a combined preservation process where osmotic dehydration is applied prior to freezing, achieves several advantages, especially in plant tissues, sensitive to freezing. OD pre-treatment can lead to the selective impregnation of solutes with special characteristics that reduce the freezing time and improve the quality and stability of frozen foods. ODF research has extensively focused on the effect of the osmotic process conditions (e.g., temperature, duration/composition/concentration of the hypertonic solution) on the properties of the osmodehydrofrozen tissue. A number of complimentary treatments (e.g., vacuum/pulsed vacuum, pulsed electric fields, high pressure, ultrasound) that accelerate mass transfer phenomena have been also investigated. Less research has been reported with regards the benefits of ODF during the subsequent storage of products, in comparison with their conventionally frozen counterparts. It is important to critically review, via a holistic approach, all parameters involved during the first (osmotic dehydration), second (freezing process), and third stage (storage at subfreezing temperatures) when assessing the advantages of the ODF integrated process. Mathematical modeling of the improved food quality and stability of ODF products during storage in the cold chain, as a function of the main process variables, is presented as a quantitative tool for optimal ODF process design.

## 1. Introduction

Osmotic dehydration (OD) involves the partial removal of water without phase change by immersing the food product in hypertonic aqueous solutions, containing carbohydrates and/or salts [1]. The main mass transport phenomena occurring during this process include the water flow from the interior of the product to the solution, the countercurrent solute transfer, and to a minor extent the product’s own solutes leaching out [2,3,4,5]. Osmotic pre-treatment aims at decreasing product water activity, without adversely affecting product characteristics as much as other dehydration (e.g., air-drying) processes. Factors that influence the rates of water removal and solute impregnation are the properties of the food material, the composition and the concentration of the osmotic solutes in the osmotic solution, the temperature of the osmotic solution, the immersion time, the level of agitation in the solution, the geometry (size and shape) of the food, and the solution-to-food ratio. Several recent publications have reported on the role of these factors on the mass exchanges during the osmotic dehydration of common fruit and vegetables, such as apples, bananas, potatoes, tomatoes, cucumbers, and so on [6,7,8,9]. Furthermore, studies have been carried out to better understand kinetics of mass exchange during the osmotic dehydration, by appropriately modeling the mechanism of the process [10,11,12,13,14]. Most of investigations used Fick’s law of unsteady state diffusion to estimate the water or solute diffusivity, simulating the experiments with boundary conditions [15,16,17].

As OD generally leads to an uptake of solutes, this process can be appropriately designed and implemented for modifying final product properties, and thus has been proposed as a tool in matrix engineering. The selective solute selection makes it possible to enrich the food product with the desired bioactive components, aiming at nutritional, textural, or flavor enhancement. The main advantages of OD are related to improved quality retention, especially when compared with other, more intense dehydration methods, such as air-drying. Especially in the case of plant matrices, such as fruits and vegetables that are prone to severe tissue damage when high temperatures are applied, OD seems a very promising technique to minimize such quality deterioration. On the other hand, according to authors in Chiralt et al., 2001 [18], the main changes induced by OD affect the texture of such tissues, mainly owing to the loss of cell turgor, the changes of the middle lamella and the cell wall resistance, and the undesirable shrinkage/change of shape and volume of the osmosed samples.

As demonstrated in several studies, osmotically dehydrated products do not reach a water activity level that would render them microbiologically stable [19]. Therefore, a complimentary preservation technique, like air-drying or freezing, is necessary for shelf life extension. The combined implementation of partial drying in a hypertonic solution (OD), followed by a conventional freezing step, is called osmodehydrofreezing (ODF). When osmotic pre-treatment is applied prior to conventional freezing, the water content decrease causes a depression of the freezing point and a significant decrease of the amount of water to be subsequently frozen. The moisture content decrease combined with a solute impregnation leads to an increase of the glass transition temperature, which makes formulated tissue much more stable during subsequent frozen storage [19,20,21,22]. As stated in Goula et al., 2012 [23], the reduction in moisture content, induced by the osmotic step, results in significant advantages, regarding the freezing process per se, including (1) improved retention of structure and other quality attributes; (2) reduced costs of packaging, distribution, and storage; and (3) energy savings during freezing of the remaining water and a decrease in the refrigeration load. Despite these well established benefits, broader exploitation, and industrial implementation of ODF is practically limited, mainly owing to difficulties in modeling and controlling mass transport phenomena, and especially owing to the problems (from an environmental, financial, and operational perspective) regarding osmotic solution management [24,25]. Finding ways of multiple re-use of the osmotic syrup is one of the most important issues regarding osmotic dehydration [26]. Another point to be considered, as pointed out in Marani et al. 2007 [27], is that the quality enhancement induced by the osmotic dehydration prior to freezing is strongly dependent on both the fruit tissue and the dehydrating used. The authors showed that, in the case of kiwis, except for drip loss, an osmotic pretreatment deteriorated faster sample quality, whereas for apples and pears, the OD step had a beneficial effect; in any case, the selection of the dehydrating osmotic solution plays a decisive role for improving quality after freezing and thawing.

**Figure 1:**
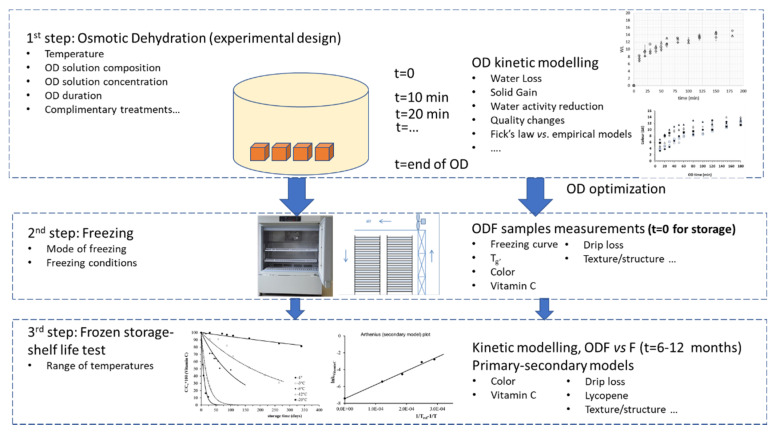
Flow chart of the distinct stages of osmodehydrofreezing (ODF), and the determining design parameters.

There are several works focusing on osmodehydrofreezing (ODF), as a means to retain frozen food quality; the majority of the published research refers to fruit and vegetable treatment, because conventional freezing methods can cause undesirable textural changes, cell rupture, and extensive softening, making this widely applied preservation technique not suitable for certain plant tissues. Besides osmotic treatment, there are other dehydration techniques that can be beneficially used prior to freezing, with air drying at different processing temperatures being the most popular [28,29,30,31]. In [32], a comparative assessment of such techniques is addressed, and alternative freezing methods are then presented. Although the quality of frozen food products versus osmo-dehydrofrozen counterparts has been previously compared, a thorough and systematic kinetic study of the most important quality indices, at a wide temperature range of frozen storage, has not been comprehensively approached. The aim of the current work is to (a) critically review recent studies on osmodehydrofreezing of food products; (b) report on different osmotic solutions applied; and (c) investigate the effect of this combined process on the quality degradation (via different quality indices) and shelf life of pretreated tissues compared with the untreated, frozen food products, through a systematic kinetic approach, covering the temperature range of frozen storage in the cold chain. The purpose is to present all aspects of this combined process in an integrated approach and indicate the necessary information that need to be retrieved in each step, in order to be able to reliably design and assess the ODF products quality during processing, storage, and distribution in the cold chain (Figure 1).

## 2. The Use of Alternative Osmotic Media in the Osmotic Dehydration Treatment, Prior to Freezing

Although air-drying has been used to remove an amount of water out of the food prior to freezing, osmotic dehydration is currently preferred as a milder technique, also offering other benefits, such as the selective food modification via the impregnation of desired solutes. When designing an osmotic treatment, the main process parameters include the temperature, solution composition and concentration, immersion time, and food/solution ratio. In James et. al. 2014 [32], a detailed table is presented that summarizes the conditions used in the case of fruit and vegetable osmotic dehydration prior to freezing. The early research in this field was based on the application of sucrose, as an osmotic agent, owing to its good performance as a water activity lowering agent. However, in more recent studies, other carbohydrates are chosen, alone or in combination to salts, in order to avoid sucrose sensorial impact and perceived negative effect on consumer health. In Table 1, representative studies using alternative carbohydrates and/or salts are listed.

Looking into more recent studies of OD application, low molecular weight carbohydrates of specific nutritional and/or functional attributes that do not significantly alter the sensory characteristics of the osmo-treated tissue are often selected over sucrose [33]. Oligofructose, applied in many cases of plant tissue (Table 1) where initial taste is not desired to be significantly altered, is a non-digestible oligosaccharide, with low sweetness, exceptional dietary fibre properties, and prebiotic activity [34]. It is recognized as a natural food ingredient and is classified as a dietary fibre in almost all European countries [35]. A high DE (dextrose equivalent = 47), low average molecular weight, maltodextrin coded as hydrodynamic mechanism (HDM) is also used for the same purpose of retaining the sensory characteristics of the raw fruit tissue, showing good performance in terms of mass transfer phenomena and water activity reduction (Table 1). Trehalose, a nonreducing disaccharide with low sweetness and little effect on blood glucose level compared with glucose, has also been used, impacting cryoprotection to membranes and proteins during freezing, when introduced in the interior of plant cells [36]. In [37] and [38], maltitol (4-O-α-d-glucopyranosyl-d-glucitol), a polyol with low molecular weight, was added in the osmotic solution, aiming at maximizing the mass transfer phenomena. Maltitol is characterized by a pleasant sweet taste; does not undergo caramelization or browning; and, owing to its stability in elevated temperatures, it is used in many baked products as well as in products of reduced calories [39]. The use of glycerol has also been reported to promote mass transfer as well as improve the quality of the final osmo-dehydrated fruits and vegetables (texture-, moistness-, and colour-retention) (Table 1). Glycerol is a low molecular weight sugar alcohol that is easily digested, non-toxic, is recognized as safe additive (EC N^o^ 1333/2008 and GRAS), and is used as a humectant to control a_w_ without % limitation.

Next to the use of different types of carbohydrates, NaCl and CaCl_2_ salts are frequently incorporated within the OD solution in small quantities for texture reinforcement purposes. Small additions of NaCl have been shown to attenuate the driving force of the mass transfer phenomena and also improve the sweet taste of products of plant origin (e.g., tomatoes in [40] and [41]). Salt concentration improves water loss at equilibrium, but shows a negative interaction effect with sucrose concentration. Salt and sucrose concentrations had a synergistic effect on soluble solid impregnation, whereas sugar concentration was shown to reduce salt gain in the fruit samples [42].

CaCl_2_ is an important factor for cell turgor retention and is often used to minimise tissue damage during processing. Its particular role is to interact with pectins and other cellular wall components, which reinforces the mechanical properties of the plant cellular matrix [18,43]. Another substance incorporated within OD solutions is the small quantities of ascorbic acid, as in the case of apricot cubes, in order to avoid undesirable enzymatic browning [19].

## 3. Mass Transport and Heat Transfer Modeling during ODF

Frequently addressed in many studies on osmotic dehydration is the mathematical description of mass transfer, expressed by the two main terms, water loss (WL) and solid gain (SG) (Equations (1) and (2)) using either empirical models or equations derived from Fick’s law. In the latter case, most published works apply the mathematical solutions of the differential equations of diffusion (Fick’s law), as presented by Crank 1975 [44] assuming specific geometries for the food in question [45,46,47,48,49]. In this case, the dehydration step is frequently described assuming that diffusion through cellular membranes and intercellular spaces is the prevailing mechanism of mass transfer [23]. A more complex model, incorporating osmosed food shrinkage and the case of samples (like mangoes) having an elevated soluble solid gradient between the surface and the center of the fruit sample, is detailed in Fernandes et al. 2019 [50].

The empirical models usually implemented include the Azuara’s model [51], which is actually a two-parameter equation, derived from mass balance considerations, and used to describe the kinetics of osmotic dehydration (water loss, WL and solid gain, SG) and the final equilibrium point. There are numerous publications in current OD literature that apply this empirical approach [49,52,53]. Another empirical model, Peleg’s, presented in Peleg 1988 [54], was initially used to fit published sorption curves, but widely applied for mass transfer modeling during the OD process [39,55]. The empirical model suggested by Page, 1949 [56] (Page’s model) is based on an exponential equation, as depicted in Table 2. Cunha et al. 2001, in [57], applied the Weibull probabilistic distribution model, stating that ‘although empirical, it is quite simple and generally gives good description of complex processes with high variability’, as the osmotic procedure of foods. Finally, in a recent study, mass transfer phenomena during impregnation of bioactive compounds from beetroot juice in apple wedges were described using a 3D geometry closely resembling the real shape of the product [58].

The above mentioned empirical equations are often combined with the use of a polynomial model to describe the main effects of the procedure parameters (namely the concentration of the osmotic solution and OD temperature) on mass transfer [46,47].

The basic equations used are presented in Table 2, and aim at calculating the following mass transfer indices (Equations (1) and (2)), namely water loss (WL) and solid gain (SG):

(1) $$WL=\frac{\left(M_{0}-m_{0}\right)-\left(M-m\right)}{m_{0}}$$

(2) $$SG=\frac{\left(m-m_{0}\right)}{m_{0}}$$

where *M*_0_ is the initial mass of fresh material, *M* is the mass after time t of osmotic treatment, *m* is the dry mass of food tissue after time t of osmotic treatment, and *m*_0_ is the dry mass of fresh material.

Some representative publications on the OD mathematical modeling for fruits and vegetables are summarized in the review of Ramya et al. 2017 [59] and in Assis et al. 2017 [10], where a comparative study of the main models presented in Table 2 is performed. Finally, in [11], a detailed presentation of available models is shown, classified by the authors into empirical, semi-empirical, phenomenological, and mechanistic, with an analysis of the advantages and disadvantages of each of them. A detailed review of these models is not in the scope of this paper, as the mathematical description of the OD process is rarely studied in publications on the ODF combined process [60].

The heat transfer mathematical description is also detailed in a few studies. In Goula et al. 2012 [23], modeling of the freezing process was carried out numerically, incorporating the phase change, via the value of enthalpy, in thermal balance equations. In another study published by Agnelli et al. 2005 [61], a model developed for the dehydrating step was coupled with the heat transfer modelling during the freezing step, applying the enthalpy formulation with a finite-volume scheme. The equations were successfully validated with experimental data obtained on pear disks and apple cubes.

## 4. Complementary Treatments to Improve Osmotic Dehydration Effects

Despite acknowledging the benefits of osmotic dehydration on quality retention, especially with regards to color, flavor, texture characteristics, and reduced drip and nutrient loss, the mass transfer rate induced merely by free diffusion of molecules is rather low, so a complementary method could be applied to accelerate the OD procedure. In the early investigations of OD, the vacuum impregnation (VI) of a porous food with an external solution was frequently used to promote fast changes of the cell membrane by the action of the hydrodynamic mechanism (HDM). Therefore, the combined application of vacuum was preferably implemented over the OD under atmospheric pressure in order to introduce the desired food ingredient directly into the product throughout its pores, in a controlled way. As far as ODF is concerned, vacuum impregnation process can assist in modifying (in a short time) the initial composition of porous fruits, introducing cryoprotectant solutes and making them more resistant to damages induced by the freeze–thawing processes. The details of this methodology, its characteristic parameters, and the changes caused to porous foods, prior to freezing, are discussed in [77]. Several publications have demonstrated the benefits of such a complimentary process, using hypertonic OD solutions, to accelerate mass transfer phenomena [71,75].

Besides the application of vacuum or pulsed vacuum osmotic dehydration (PVOD), another novel process is the application of ultrasound, which produces a cavitation phenomenon and can generate sponge effect owing to the formation of internal microscopic channels in the cell tissue [78]. Consequently, the use of ultrasound in osmotic dehydration aims at improving mass transport rates, through a modification of the cell membrane permeability. In [79], kiwifruit slices were submitted to osmotic dehydration using a 50% sucrose solution, with different ultrasound power (20 kHz, 0–300 W) at 30 °C for 30 min, and then frozen as well as stored at −18 °C. The results showed that the application of ultrasound can effectively increase the freezing rate and improve fruit physicochemical properties in freezing and thawing processes. The same conclusions were drawn in another survey, where red radish cylinders were immersed in 60% sucrose solution, with ultrasound simultaneous application (40 kHz, 40 kHz−250 W) [80]. Similarly, the ultrasound assisted osmotic dehydration was found to shorten the dehydration time and better preserve the firmness and L-ascorbic in broccoli [66].

On the basis of the same principles, pulsed electric fields (PEFs) processing is used to increase the permeability of the cell membrane and can accelerate mass transport out of the cells, leaving the product matrix largely unchanged. The application of pulsed electric fields (PEF) in combination to OD was tested as a prefreezing step in Fan et al. 2020 [79] for kiwifruit, using a ternary OD solution, containing glycerol (30%), high-DE (dextrose equivalent) maltodextrin (20%), trehalose (10%), ascorbic acid (2.0%), calcium chloride (1.5%), sodium chloride (1.0%), and citric acid (0.2%) (*w/w*). The authors concluded that PEF and OD could lead to adequate mass transport, satisfactory a_w_ decrease, and improved quality characteristics of processed kiwifruit. The same complimentary treatment (PEF) was also used in [69], where apple discs were immersed in solutions containing apple juice-glycerol osmotic solutions with different glycerol concentrations, 0–60 wt. %, leading to noticeable acceleration of the freezing/thawing processes as compared with those for untreated samples.

The application of OD and high pressure (HP) technologies has been also investigated to optimize the mass transfer as well as the quality attributes of the osmosed plant tissues [81,82,83,84]. When food is pretreated with high hydrostatic pressure (HP; 100–800 MPa), it also results in disintegration of cells [85,86]. In Dash et al. 2019 [84], the authors studied the osmotic dehydration of ginger slices in three different osmotic agents (sucrose, fructose, glucose, 60 °Brix, 40 °C, 0–180min) after HP application (0.1–600 MPa, −40 °C for various times). They observed that increased pressure enhanced WL and SG. HP processing at 600 MPa increased the effective moisture diffusivity values by approximately 11–12-fold. Among the osmotic agents tested, solute diffusivity was maximum for glucose followed by fructose and sucrose, whereas the moisture diffusivity was greatest for sucrose and lowest for glucose. In Dermesonlouoglou et al. 2017 [87], HP (600 MPa−25 °C−5 min) processed osmotically dehydrated strawberry cubes (35 °C, −45 min) (OHP) were subsequently stored at isothermal and dynamic temperature conditions. They conducted a full and systematic kinetic study of important quality indices (microbial growth, sensory tests, texture, color, anthocyanins, total phenolic content). They concluded that the shelf-life of OHP strawberry cubes was extended to 12 months when stored at 4 °C (microbiologically stable products throughout chilled storage, at the same time maintaining their quality and sensory characteristics), while the respective shelf life of OD treated samples at 4 °C was 115 days, limited by microbial growth.

## 5. Quality Degradation during Frozen Storage of ODF Products: Kinetic Modeling of Important Quality Indices

Although there are numerous studies on the application of OD, using different osmotic media, different process conditions, and applying different equations to describe the mass transfer phenomena, limited research has been performed on the subsequent freezing method used [32,67,74], and especially on the quality degradation rates of the treated versus the untreated food product, in a wide range of freezing storage temperatures. In James et al. 2014 [32], the authors report the few studies that describe the exact conditions of freezing, with the majority applying conventional methods, such as air freezing, at −40 °C. Another finding when reviewing the relative literature is that most studies investigate the quality status of both OD pretreated and untreated frozen samples immediately after the freezing process, without assessing OD benefits throughout food storage at freezing conditions. In [88], several physicochemical indices of osmotically pretreated strawberries (with sucrose) were measured after a 5-month storage at −18 °C, testing the influence of OD processing time on the final frozen product. Their conclusion was that samples treated for 1–2 days at 5 °C could be directly consumed after thawing in the substitution of fresh fruits.

There are only a few studies that monitor sample quality deterioration during frozen storage, and thus perform a systematic kinetic study in order to estimate rates of degradation at different temperatures, instead of single values of quality parameters.

In this section, the main quality indices measured are briefly described, the most frequently used mathematical models to describe quality change over storage time (primary model) are reported, and any information on product shelf life (or other kinetic info that could serve for shelf life estimation) is discussed. In summary, Table 3 is presented as an overview of the studies that provide such information, which can be further exploited to comparatively assess food shelf life, with and without OD pretreatment. The advantage of shelf life estimations is that the established knowledge of the beneficial application of OD prior to freezing on frozen food quality can be quantified and better explained.

As far as food status immediately after the freezing process is concerned, several studies have reported the depression of the freezing point obtained with OD pretreatment, owing to the water activity reduction. This approach, focusing on the negative effect of large ice crystals on the drip loss and softening of thawed plant tissues, aims at illustrating the benefits of shortening the freezing time on the quality characteristics of the thawed matrix [66]. In [66], the freezing time of broccoli was reduced from 19.8 min (untreated samples) to 10 min (for the OD-frozen and the ultrasound-OD-frozen samples), leading to improved texture characteristics of the treated tissue. Tomato cubes were osmotically pretreated with maltodextrin in [67] prior to cryogenic freezing and the freezing time was reduced from 4.48 to 3.13 min.

Another important factor that affects quality degradation is the glass transition temperature of the maximally concentrated phase (T_g_’), which is not often measured in the osmo-treated tissue. Below this temperature range, degradation processes that are diffusion-controlled are significantly detained in the glassy state, resulting in limited food deterioration [38]. Bearing in mind the improved stability obtained when perishable foods are stored below or close to that zone, it is important to assess the OD effect on the glass transition [89]. Osmotic pretreatment in carbohydrate solutions, leading to tissue modification owing to water loss and solid impregnation, increases the value of T_g_’ and could improve the frozen food quality retention, especially when temperatures throughout the chill chain do not deviate from the proper ones. Such an approach was followed in Giannakourou and Taoukis 2003 [38], where samples treated with oligofructose (Table 2) were found to have the highest Tg’. In another study [90], mango cuboids were subjected to OD in mixed sugars, with sucrose, glucose, and fructose in a ratio of 3.6:1:3, and then stored at −55 °C in a glassy state. By measuring several physicochemical properties for up to 6 months, the authors concluded that the quality of frozen mangoes continued to change, even in the glassy state; however, the quality changes of the osmo-dehydrated samples were less pronounced than those of the untreated samples. Similarly, the T_g_’ of pre-treated rambutan pieces with sucrose, trehalose, and maltitol syrups was found to be significantly increased in [37]. In this study, the quality of frozen samples was assessed after 120 days of storage at −18 °C, a temperature that remained within the rubbery state. Therefore, it is worth continuing to investigate the role of T_g_’ increase per se, even if a glass temperature value of the treated matrix within the glassy state is not obtained. There are published data showing that the stability of quality parameters, such as color and vitamin C, was better retained when the temperature of frozen storage was close to the glass transition temperature of the modified food product [38,91].

Regarding kinetic studies under frozen storage conditions, it has been reported that osmotic dehydration before freezing decreased the rates of degradation of physiologically active components such as vitamins and minerals, especially in the case of vulnerable tissues, such as fruits and vegetables. The color of pretreated frozen samples was better retained, and the texture characteristics of thawed fruits and vegetables were superior compared with those of the untreated matrices. Finally, other indices measured during shelf life tests included drip loss.

### 5.1. Shelf Life Indices of Osmodehydrofrozen Foods

The main quality factors investigated during frozen storage of osmodehydrofrozen samples, depending on the raw product, are briefly described.

#### 5.1.1. Vitamin C

Ascorbic acid is a quality index, frequently measured in frozen fruit and vegetables studies. When blanching is not effectively applied in plant tissues, the presence of ascorbate oxidase catalyzes oxidation of L-ascorbic acid during storage, a process significantly accelerated when oxygen-permeable packaging is used. The rate of oxidation depends mainly on storage temperature and product pH [92]. In most published studies, vitamin C loss is found to adequately follow an apparent first-order reaction, Equation (3) [93], and temperature dependence is expressed by the Arrhenius equation (Equation (4)).

(3) $$C_{Vit}=C_{Vit_{o}}e^{-k_{vit}t}$$

where *C_vito_* is the initial concentration of vitamin C (mg/kg), *k_vit_* is the rate of vitamin C loss:

(4) $$k=k_{ref}\left[\frac{-E_{a}}{R}\left(\frac{1}{T}-\frac{1}{T_{ref}}\right)\right]$$

where *E_a_* is the activation energy of the chemical reaction, *k_ref_* is the reaction rate at the reference temperature *T_ref_*, and *R* is the universal gas constant.

As demonstrated in Table 3, the application of an OD pretreatment significantly stabilized L-ascorbic acid against oxidation. This could be attributed to water activity reduction, or to a T_g_’ increase, accomplished by water loss and solid impregnation. The incorporation of molecules such as oligofrucrose, trehalose, or maltitol into the OD solution seems to be more effective in vitamin C retention in frozen tissues [33,38]. As discussed in Fito et al. 2001 [94], the glass transition increase accomplished through an osmotic step could increase the fruit stability during frozen storage, also confirmed in [19], although the beneficial effect of sorbitol observed in apricot cubes in this study, could not be explained solely through the glass transition theory. As already pointed out, further research is needed to define the integrated impact of different factors such as pH, water content, freeze-concentration, and specific properties and characteristics of the carbohydrate used in OD solutions, influencing this vitamin degradation. In Xin et al. 2014 [66], it was demonstrated that the application of a complimentary procedure, namely ultrasound, made the positive effect of osmotic dehydration more pronounced, regarding vitamin C retention during frozen storage (at −25 °C for 6 months).

Although the rates of degradation are reduced during frozen storage for OD samples (compared with the untreated ones), it should be pointed out that the ascorbic acid content of products decreased in part after the osmodehydrofreezing process, possibly owing to a mild leakage of water-soluble compounds out of the cell tissue [80]. Zhao et al. 2017 in [90] showed that the osmotic pretreatment with a mixture of sucrose/glucose/fructose significantly decreased the vitamin C content of ODF mangoes before frozen storage (with OD samples having a water content of approximately 80% w.b.). This initial loss was counterbalanced by the improved performance of OD samples towards vitamin C retention during subsequent storage, especially after 3 months, possibly owing to rapid leaching via increased drip loss of the untreated samples. Fan et al. 2020 [79] studied the application of osmotic dehydration with ultrasound enhancement before freezing of kiwi and showed a better retention of ascorbic acid content; however, they stressed that, because ascorbic acid is water-soluble, it can be easily lost during thawing, thus the extent of drip loss in ODF samples is strongly related to the retention of ascorbic acid. These results are in agreement with Xu et al. 2014 [80], who found that the ascorbic acid content of radish cylinders decreased after the osmodehydrofreezing and thawing process, owing to the leakage of water soluble vitamin C from the interior of product cells. Similar findings can be found in Xin et al. 2014 [66], who measured a lower loss of L-ascorbic acid into the osmotic solution (containing 40% trehalose) when an ultrasound-step was applied, mainly attributed to the shorter dehydration time compared with the OD counterparts (with a water content of approximately 65% w.b.).

**Figure 2:**
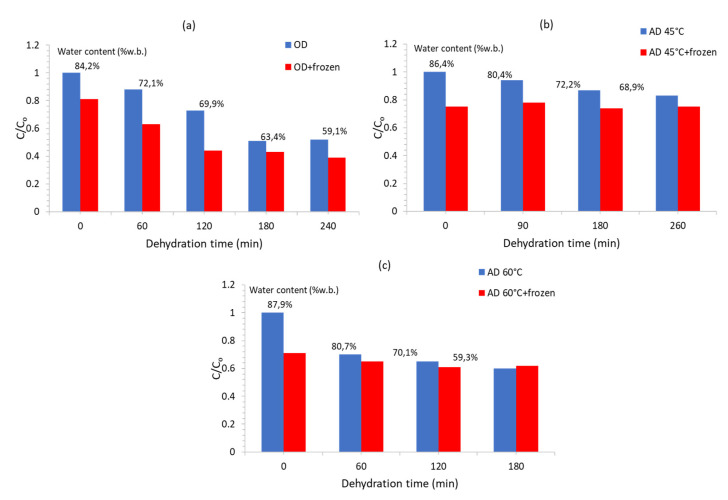
Vitamin C retention in pineapple slices during (**a**) osmotic dehydration at 45 °C–60 °C Brix and (**b**) air drying (AD) at 45 °C and (**c**) air drying (AD) at 60 °C prior to freezing and after the freezing/thawing process. (data adopted from [28]). Percentages presented above the bars indicate the respective water content of samples (expressed on a % wet basis).

Concerning the effect of OD versus other dehydration pretreatments prior to freezing (immediately after processing, before any subsequent storage) on the nutritional losses of fruits and vegetables, there are few published data that allow for such a comparative assessment. In Ramallo and Mascheroni 2010 [28], osmotic dehydration and air drying were applied on pineapple before freezing and the vitamin C content decrease was measured (Figure 2) on dehydrated as well as dehydrofrozen samples. The results showed that ascorbic acid loss during OD was more extensive than during air drying, even at the highest temperature of 60 °C. Nutritional degradation of all samples is further increased after freezing–thawing, with ODF showing a retention only near 40%. One should further investigate the role of water content in the dehydrated tissue (respective values of water content are included within the figures), as well as the effect of the amount and the type of solutes incorporated. In Robbers et al. 1997 [95], osmotic and convective dehydrofreezing were studied on kiwi samples and the time needed to reach the desired moisture (final water content, 40–50–60% w.b.) was found to be reduced with combined drying compared with osmotic drying. As far as L-ascorbic acid loss, it was more intense in the convective air drying process, especially at high temperatures and extended drying times. The addition of ascorbic acid into the osmotic solution substantially prevented notable vitamin C loss during OD and, in some cases, an increase in ascorbic acid was measured.

#### 5.1.2. Color

Frozen products of plant origin undergo various modifications in color during storage, owing to changes in the naturally occuring pigments, such as chlorophylls, anthocyanins, and carotenoids, or by enzymatic browning [92]. The characteristic green color of frozen peas, green beans, and spinach gradually turns to brown during storage at −18 °C, owing to the transformation of chlorophyll a and chlorophyll b into their corresponding pheophytins. Such changes in the initial color occur much more quickly in unblanched vegetables or those stored at insufficiently low temperatures. Another alternative path of chlorophyll breakdown is by the action of lipoxygenase, which causes an oxidation of polyunsaturated fatty acids in the presence of oxygen. The rates of color degradation owing to chlorophyll tranformation vary significantly depending on the tissue in question and its particular physicochemical characteristics [96].

When color change is related to fruits with red hue, this is because of anthocyanin (water soluble) destruction by enzyme-induced oxidation of the polyphenols during processing and storage. Additionally, the oxidation of carotenoids (liposoluble pigments, present in many plant tissues in the form of xanthophylls), carotenes, and lycopene is another source of modifications in color; however, blanching, a necessary pretreatment for frozen vegetables, significantly inhibits such degradations, preserving both chlorophyll and carotenes, the latter acting as provitamin A.

Alterations in color owing to enzymatic browning are caused by the oxidation of phenols in the presence of oxygen in products with a white or light yellow color, such as mushrooms, apples, potatoes, fish flesh, and so on. Polyphenol oxidases act as catalysts for this reaction, leading to the formation of quinones that condense in the form of brown or reddish-brown compounds. Subsequently, the quinones act as oxidants for other substrates such as ascorbic acid, anthocyanins, and so on. The undesired development of brownish color may be effectively hindered by blanching, the addition of chemical inhibitors (e.g., ascorbic acid, SO_2_, Na_2_SO_3_, and so on), or the exclusion of oxygen, by an appropriate packaging.

In most cases, in frozen food stability studies, the instrumentally measured color is assessed in a L, a, and b scale, depicting the visual change observed, instead of investigating the real underlying mechanism (e.g., anthocyanin or phenol oxidation). Therefore, color modifications may be described by changes in either L (when lightness is the main property altered), a, and/or b parameters when there is a nuance change, or more complex color expressions, such as ΔC (chroma value) or ΔE (total color change, taking into account all color parameters):

(5) $$\Delta C=\sqrt{{\left(a-a_{0}\right)}^{2}+{\left(b-b_{0}\right)}^{2}}$$

(6) $$\Delta Ε=\sqrt{{\left(L-L_{0}\right)}^{2}+{\left(a-a_{0}\right)}^{2}+{\left(b-b_{0}\right)}^{2}}$$

where L, a, and b are the instrumentally measured colour (CIELab) values and the subscript ‘o’ denotes initial values, at time zero. In most shelf life studies of ODF samples, temperature dependence is expressed by the Arrhenius equation (Equation (4)).

#### 5.1.3. Structure and Texture

Food texture is a critical quality factor strongly influenced by freezing. Texture can de described by the rheological and structural properties of the food. When studying the texture of frozen fruits and vegetables, the main attributes measured include firmness (or hardness), tenderness, and crispness. Those characteristics are related to both the structure and the turgor of the cell, as well as the rigidity provided by the cell wall [97]. Owing to ice-crystal growth and all underlying phenomena (ice nucleation, ice crystal size induced by the freezing process, ice crystal location, and so on), the texture of frozen and thawed plant tissues is bound to degradate; thus, there has been increased interest in investigating the effects of ice crystal size and location, as well as tissue swelling on the structure of frozen foods. A lot of research is focused on ways to maximize texture retention, and, in this context, the moisture reduction, and the selective incorporation of ingredients within the plant cell obtained through ODF has been proven to be an attractive technique. Water is drawn out of the cells owing to the osmotic pressure gradient, leading to a freezing point decrease and a reduced fraction of ice at a given frozen storage temperature. There are several studies that show that OD application prior to freezing can reduce the structural collapse after thawing–rehydration of fruits and vegetables [37,98]. Furthermore, calcium salts may be incorporated into fruits, such as strawberries, to improve firmness, with the addition of pectinmelthylesterase, maximizing this beneficial effect [1]. Fruits such as strawberries, apricots, cherries, kiwi, and pineapples have been subjected to osmotic drying prior to freezing, producing final products with lower drip loss and improved firmness [99]. In Erba et al. 1994 [100], apricot and peach cubes osmodehydrated in fructose sorbitol syrup were found to be softer than the fruits osmodehydrated in fructose, producing dried products, possibly consumed as snacks or as ingredients in foods such as pastry, ice cream, and so on.

However, the changes in mechanical properties caused by the OD treatment itself should not be overlooked. Careful process conditions should be applied if texture retention is a main target, apart from the desired improved color, flavor, and vitamin, during frozen storage. In [99], it is stressed that OD duration should be selected taking into account not only the overall water content reduction, but also the even distribution of this component. In the same study, the negative effect of vacuum pulse owing to the excessive drip loss has also been mentioned.

**Figure 3:**
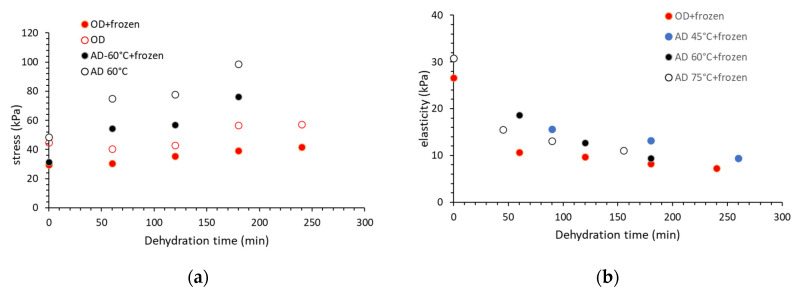
Mechanical properties change during dehydration of pineapple slices, expressed by (**a**) stress increase during osmotic dehydration at 45 °C–60 °C Brix and air drying (AD) at 60 °C prior to freezing and after the freezing/thawing process and (**b**) elasticity decrease during osmotic dehydration at 45 °C–60 °C Brix and air drying (AD) at 45, 60, and 75 °C after the freezing/thawing process (data adopted from [28]).

When studying the effect of OD versus other dehydration pretreatments prior to freezing on the texture characteristics of plant tissues, there are some interesting published data in Ramallo and Mascheroni 2010 [28], concerning pineapple dehydrofreezing. The authors measured the stress at fracture, and the elasticity of fresh and dehydrated samples, before and after the freezing–thawing process (Figure 3). These mechanical properties are a representative index of samples firmness and system elastic resistance. The results showed that the failure stress of pineapple samples after air-drying was greater than after OD, with equal moisture content (around 60% w.b.), and compared with the fresh fruit. These findings reveal that products obtained by osmotic treatment are firmer than their counterparts of fresh fruit, but are softer than those obtained by hot air. As far as the freezing–thawing process is concerned, it leads to a clear decrease in fracture stress values for all dehydrated pineapple samples, compared with samples immediately after dehydration (Figure 3a). The hardness of pineapple samples dried by hot air was similarly affected by the freezing–thawing process than those dehydrated by OD, with a similar moisture content. Next to the stress increase, both air drying and OD cause a decrease in elasticity (Figure 3b) and the authors conclude that the changes observed suggest that no cellular debonding occurs owing to these dehydration processes. In any case, it is evident that the freezing–thawing procedure, followed by cellular damage owing to ice crystal formation, leads to turgor loss of pineapple tissue, which is more pronounced in untreated and OD-treated samples.

Regarding texture retention during frozen storage, the shelf life study showed that osmodehydrofrozen compared with conventionally frozen sliced cucumbers, strawberries retained their firmness for prolonged storage period [33,60]. In [40], it was demonstrated that, after a year of storage, the firmness of samples pretreated with high dextrose equivalent maltodextrin (HDM), oligofructose, and the mixture of oligofructose/trehalose was about 18–75% higher than the corresponding values of the untreated tomato samples, although, in all cases, texture was observed to rapidly degradate. Similarly, in [72], the protective effect of OD pretreatment on watermelon cell integrity during the frozen storage was shown, when glucose, oligofructose, and HDM, along with CaCl_2_ were included in the OD solution, prior to freezing. In this case, calcium chloride also contributed to the increased firmness owing to the interaction with the cellular wall components of the plant matrix, an observation also mentioned in [90]. In the same study, it was also observed that the initial texture softening immediately after OD was counterbalanced by a much improved quality retention during the subsequent long period frozen storage.

#### 5.1.4. Drip Loss

Another important issue regarding the quality deterioration of frozen foods is related to extensive loss of liquids, or drip loss, when thawed. Not all frozen foods have to undergo a thawing procedure prior to heat treatment or final consumption. In the case of frozen fruits, products of animal origin, and so on that are submitted to thawing, thawed food items should be treated carefully, taking measures such as appropriate cleaning and disinfecting of food contact surfaces, in order to avoid possible microbial contamination. Therefore, it is important to investigate ways to decrease the liquid leakage (drip loss) from the thawed tissue, in order to also avoid loss of water-soluble substances. As partial removal of water, especially from plant tissues, induces the concentration of cytoplasmatic components, the depression of the freezing point, the increase of supercooling, and microcrystallization, as well as a significantly lower proportion of ice crystals to the unfrozen phase, damage of cellular integrity as well as drip loss, caused by freezing, is expected to be substantially decreased. In [101], it was found that papaya samples, pretreated with 45 and 55% sucrose for 90 min (at room temperature) exhibited lower drip loss with respect to control samples, when stored at −40 °C for a period of up to 60 days. The beneficial effect of OD pretreatment on thawed broccoli and radish drip loss was also confirmed in [66] and [80], respectively, especially when ultrasound was additionally applied. Similar results were obtained in [102,103] and [1,33,90] for ODF melon, cantaloupe, mangoes, and strawberries, respectively.

#### 5.1.5. Chlorophyll, Lycopene, Anthocyanins, and Other Pigments

As already discussed for color loss, this quality degradation is mainly attributed to different pigments oxidation, which does not cease to occur during frozen storage. Next to instrumentally measured color change, pigments’ gradual loss is measured in few studies of ODF products of plant tissues. In all of these studies, the incorporation of solutes combined with a water decrease following the OD process was found to improve pigment retention, in a similar way in which vitamin C was found to be better preserved. On the basis of the glass-transition concept, the observed stability can be related to the increased viscosity and the limited molecular mobility of the unfrozen phase, mainly owing to the glass-transition temperature increase obtained. However, this theory needs a careful approach as many chemical reactions are not diffusion controlled. The temperature difference between the T_g_’ and the storage temperature is found to play an important role in tissue stabilization, thus manipulation of the OD composition could affect this temperature difference and the observed reaction rates [104]. It is worth mentioning that, in [105] and [106], it was shown that the abovementioned general theory is not valid for all pigments; in those studies, it was demonstrated that, for anthocyanin in strawberry and blueberry, a simple relationship does not exist between the degradation of these phytonutrients and the range of the difference between the storage temperature and T_g_’. The authors commented on this finding, stressing the role of other factors, such as the pH of the unfrozen state or the nature of the osmotic agent used. On the other hand, this kinetic interpretation based on the glass-transition temperature seems to be confirmed for lycopene in ODF watermelon tissues [65]. In [41], lycopene loss was found to be delayed during frozen storage of tomato slices, and a possible explanation, according to authors, is the incorporation of sugar into food matrix, which strengthens the binding force of lycopene in the tomato tissue, also hindering oxygen penetration and thus lycopene oxidation.

#### 5.1.6. Sensory Attributes

In [33,60,70], a systematic sensory evaluation was also conducted in parallel to instrumental measurements, in order to correlate analytical results of texture and color with the respective sensory evaluation. Panel assessments confirmed the analytical results of better quality retention of ODF cucumbers, strawberries, and kiwi, also judging their flavour as ‘pleasant and organoleptically acceptable’. On the other hand, regarding frozen tomatoes, the positive effect of the osmotic pretreatments is more pronounced during subsequent frozen storage [40] than immediately after freezing of OD samples.

### 5.2. Shelf Life Modeling during Frozen Storage of ODF Food

In most cases in Table 3, where quality was assessed in isothermal conditions, the effect of temperature on quality change rate is modeled by an Arrhenius equation (Equation (4)).

Another secondary model applied in [38], the Williams-Landel_Ferry (WLF) model, in conjunction with the Arrhenius equation, is as follows:

(7) $$\log\frac{k}{k_{\mathrm{ref}}}=\frac{C_{1}\left(T-T_{\mathrm{ref}}\right)}{C_{2}+\left(T-T_{\mathrm{ref}}\right)}$$

where k_ref_ is the reaction rate at the reference temperature and C_1_ and C_2_ are coefficients, which, unlike the Arrhenius equation’s ‘activation energy’ (Equation (4)), do not have an obvious or direct kinetic interpretation because they depend not only on the material being studied, but also on the chosen reference temperature [107,108]. In the specific research, an alternative to the Arrhenius secondary model was used, because in the case of oligofructose and the mixture of oligofructose/trehalose (carbohydrates used in the OD solution), a single Arrhenius plot could not adequately describe the kinetics over the whole temperature range studied (−3 to −24 °C). A “break” point was observed in the Arrhenius plots (where ln(k_ref_) is plotted vs. 1/T) between −16 and −18 °C, probably corresponding to the increased T_g_’ value of the OD pre-treated matrices, an assumption supported by the relative DSC measurements performed.

On very few occasions, the developed kinetic models (derived based on isothermal data) were further validated under fluctuating conditions [70].

When observing shelf life predictions in Table 3, it is evident that the application of OD prior to freezing could be an effective technique to increase food shelf-life, as an improved durability of more than twofold compared with untreated samples was observed in most cases. This is particularly important to mitigate food spoilage and contribute to the food chain sustainability when there is an ever-increasing demand for food that follows the exponential growth world population [109].

## 6. Discussion

Osmodehydrofreezing, described as an attractive alternative to the traditional freezing process, is found to offer numerous advantages, especially when applied to fruits and vegetables, that are prone to be severely damaged during freezing. Recently, research in the field of osmotic dehydration (first step of the integrated procedure of ODF, Figure 1) is mainly focused on the use of a variety of carbohydrates, salts, and other compounds in the osmotic solution; the complimentary application of treatments to increase mass transfer phenomena; and the more sophisticated modeling of the mass exchange process. Upon reviewing the literature on ODF, the main concern is to assess OD benefits on product physicochemical, quality, and mechanical characteristics, immediately after freezing. Little attention is given to the freezing process itself, and the significant importance of the method/equipment used and the freezing rate obtained. Surprisingly, although the freezing process is a key-stage of the whole procedure, there are rare publications that applied novel rapid freezing techniques, such as cryogenic, high pressure assisted, and so on (second step). Additionally, few studies have addressed the issue of osmodehydrofrozen versus frozen food stability during subsequent frozen storage in a wide range of temperatures, applying mathematical modeling of the quality determining indices. Another weak point in those studies is related to a well justified explanation of quality retention during storage (third stage of Figure 1) in terms of glass transition theory and freezing point depression, associated with the use of the specific OD solutions and process conditions. This holistic approach would be very useful in order to better understand the benefits of the pretreatment on the quality of the final products, so as to be able to optimize both the OD step and the freezing process itself. Another final remark is that findings from the relative studies, even if adopting an integrated approach, cannot be compared in a straightforward way, because almost all parameters involved (e.g., food matrix studied, OD conditions, carbohydrates selected) are different in each investigation. Consequently, it is hard to distinguish the distinctive role of each step and its related parameters, so as to be able to select the optimum conditions, based on the maximization of quality retention of the ODF products. It is to be hoped that these key issues will be further addressed in future studies, allowing for evaluating the ODF process as an integrated ensemble in the effort of improving frozen food quality during storage.

## Figures and Tables

**Table 1:** Carbohydrates and salts (types and concentrations) used in osmotic solutions in representative studies on osmodehydrofreezing. OD, osmotic dehydration.

Carbohydrate/Salt	Product, (Concentration (% W.T), OD Temperature and Duration)	Reference
Glucose	Koho grape (40%, 20 °C, 60 min)	[62]
	Mangoes (45%, 30 °C, 2 h)	[63,64]
	Strawberry (56.5%, 35 °C, 150 min)	[33]
	Tomato (56.5%, 35 °C, 60 min)	[40]
	Watermelon (50%, 35 °C, 60 min)	[65]
Trehalose	Koho grape (40%, 20 °C, 60 min)	[62]
	Broccoli (40%, 35 °C, 120 min)	[66]
	Rambutan (50%, 30 °C, 5 h)	[37]
Lactose	Koho grape (20%, 20 °C, 60 min)	[62]
Maltodextrin	Tomato cubes (55%, 35 °C, 24 h)	[67]
	Marinated gilthead seabream (40%, 15 °C, 6 h)	[68]
	Strawberry (56.5%, 35 °C, 150 min)	[33]
	Cucumber (56.5%, 15 °C−35 °C−55 °C for 360, 300, and 180 min, respectively)	[60]
	Tomato (56.5%, 35 °C, 60 min)	[40,41]
	Watermelon (50%, 35 °C, 60 min)	[65]
Glycerol	Apple slices (0, 20, 40, 60 wt. %, 180 min)	[69]
	Apricot (40–60%, 25–45 °C, 0–240 min)	[47]
	Cucumber (50%, 35 °C, 90 min)	[9]
	Peach (40–60%, 25–45 °C, 0–240 min)	[46]
	Kiwifruit (30%, 35–55 °C, 0–240 min)	[70]
	Tomato (50%, 35 °C, 90 min)	[9]
Sorbitol	Apple slices (50 wt. %, 50 °C, 40 min)	[71]
	Apricot (65%, 45 °C, 120 min)	[19]
Maltose	Mangoes (45%, 30 °C, 2 h)	[63,64]
	Apricot (65%, 45 °C, 120 min)	[19]
High fructose corn syrup (HFCS)	Strawberries (50%, 25 °C, 45 min, vacuum pressure of 50 mmHg)	[72]
Oligofructose	Strawberry (56.5%, 35 °C, 150 min)	[33]
	Cucumber (56.5%, 15 °C−35 °C−55 °C for 360, 300, and 180 min, respectively)	[60]
	Tomato (56.5%, 35 °C, 60 min)	[40]
	Watermelon (50%, 35 °C, 60 min)	[65]
	Green peas (56.5%, 35 °C, 4 h or 5 °C, 12 h)	[38]
Maltitol	Rambutan (50%, 30 °C, 5 h)	[37]
	Green peas (56.5%, 35 °C, 4 h or 5 °C, 12 h)	[38]
Calcium lactate	Mangoes (1%, 25 °C, 1 h)	[73]
	Papaya (1.5%, 45 °C, 4 and 8 h)	[74]
Calcium gluconate	Papaya (1.5%, 45 °C, 4 h)	[74]
Mixture of calcium gluconate and calcium lactate	Strawberries (12%, 25 °C, 45 min, vacuum pressure of 50 mmHg)	[72]
NaCl/CaCl_2_	Tomato (3.5 and 1.5%, 35 °C, 60 min)	[40,41]
	Cucumber (3.5 and 1.5%, 15 °C−35 °C−55 °C for 360, 300, and 180 min, respectively)	[60]
Ascorbic acid	Apricot (1%, 45 °C, 120 min)	[19]
	Apple (1%, 25 °C, 30 min, vacuum pressure of 100 mbar)	[75]

**Table 2:** Main equations of mathematical description of mass transfer phenomena during osmotic dehydration (as a preliminary step to subsequent freezing).

Model Name	Mathematical Expression	Explanation of Symbols	Reference
Azuara	$\frac{t}{WL}=\frac{1}{s_{1}*WL_{\infty }}+\frac{t}{WL_{\infty }}\;\frac{t}{SG}=\frac{1}{s_{2}*SG_{\infty }}+\frac{t}{SG_{\infty }}$	WL_∞_ and SG_∞_ are the terms that express the water loss/solid gain at equilibrium, and s_1_ and s_2_ are parameters that can be defined as relative rate constants for water loss and solids gain, respectively	[51]
Crank (based on Fick’s second law for diffusion)	For spherical shape: $MR=\frac{\left(M_{t}-M_{\infty }\right)}{\left(M_{0}-M_{\infty }\right)}=\sum_{n=0}^{\infty }\left(\frac{6}{n^{2}\pi^{2}}\right)exp\left(\frac{D_{ew}\pi^{2}}{r^{2}}n^{2}t\right)\;SR\frac{\left(S_{t}-S_{\infty }\right)}{\left(S_{0}-S_{\infty }\right)}=\sum_{n=0}^{\infty }\left(\frac{6}{n^{2}\pi^{2}}\right)exp\left(\frac{D_{es}\pi^{2}}{r^{2}}n^{2}t\right)$ For slabs $\frac{\left(M_{t}-M_{\infty }\right)}{\left(M_{0}-M_{\infty }\right)}=\frac{8}{\pi^{2}}\sum_{n=0}^{\infty }\frac{1}{{\left(2n+1\right)}^{2}}exp\left[-\frac{\pi^{2}{\left(2n+1\right)}^{2}D_{ew}}{4L^{2}}t\right]$ $\frac{\left(S_{t}-S_{\infty }\right)}{\left(S_{0}-S_{\infty }\right)}=\frac{8}{\pi^{2}}\sum_{n=0}^{\infty }\frac{1}{{\left(2n+1\right)}^{2}}exp\left[-\frac{\pi^{2}{\left(2n+1\right)}^{2}D_{es}}{4L^{2}}t\right]$	MR and SR are the diffused moisture and solute ratio, respectively; M and S are the moisture and solute content, respectively; the subscripts o, t, and ∞ represent the relevant values at time 0, t, and at equilibrium, respectively; D_ew_ and D_es_ (m^2^/s) are the effective coefficients of water and solute diffusivity, respectively; r (m) is the average radius of sphere; and L(m) is half the thickness of a slab (when OD occurs in both sides of the slab)	[44]
Peleg	$M=M_{0}\pm \frac{t}{k_{1}^{\prime }+k_{2}^{\prime }*t}\;\mathrm{WL}\;\mathrm{or}\;\mathrm{SG}=\frac{t}{k_{1}+k_{2}*t}$	M is the moisture at time t and M_0_ is the initial moisture (both in dry weight basis). The moisture sorption curves approach the equilibrium asymptotically using the parameters k1’ and k2’. $\left|M-M_{o}\right|$ is considered to be WL and SG, k_1_ and k_2_ are the Peleg’s constants for WL and SG.	[54]
Page	$\frac{WL}{WL_{\infty }}\mathrm{or}\;\frac{SG}{SG_{\infty }}=1-\exp\left(-A*t^{B}\right)$	A and B are Page’s constants.	[56]
Weibull	$\frac{WL}{WL_{\infty }}\mathrm{or}\;\frac{SG}{SG_{\infty }}=1-\exp\left[-{\left(\frac{1}{\tau }\right)}^{\beta }\right]$	τ is the scale parameter of the Weibull’s mode that is associated with the process rate (the time required for $\frac{WL}{WL_{\infty }}$ or $\frac{SG}{SG_{\infty }}$ to reach the value 1-e^−1^), and β is the shape parameter.	[57]
Polynomial model	$Y=a_{0}+\sum a_{i}X_{i}+\sum a_{ii}X_{Ι}^{2}+\sum a_{ij}X_{i}X_{j}$	Y represents the selected response variables (for example, WL, SG, a_w_, quality attributes measured during OD) and Xi are the factor variables [i.e., the process parameters, for example, OD solution concentration, process temperature and time, and so on] α_ο is_ is the constant, α_i_ represents the linear, α_ii_ the quadratic, and α_ij_ is the interaction effects of the factors.	[46,47,76]

**Table 3:** Comparative kinetic studies for quality degradation of osmodehydrofrozen foods versus conventionally frozen during frozen storage.

Food Product	Quality Index	Storage Temperature	Primary Model	Shelf Life Calculations Based on Kinetic Information Provided	Reference
Kiwifruit (untreated, OD, and PEF-OD)	Vitamin C (in mg L-ascorbic acid/100 g fresh material)	−5, −10, −15, and −25 °C	First order $C=C_{0}e^{-k_{vitC}t}$	Shelf life at −18 °C (untreated, OD, and PEF-OD): 200, 450, and 500 days	[70]
	Color		First order $\frac{\Delta {Ε}_{max}-\Delta Ε}{\Delta {Ε}_{max}}=\exp\left(-k_{colour}*t\right)$ (where ΔE is expressed by Equation (6))	Shelf life at −18 °C (untreated, OD, and PEF-OD): 90, 540, and 400 days	[70]
	Overall sensory acceptance (scoring in a 1–9 descriptive intensity scale)		Zero order $S=S_{0}-k_{sensory}*t$	Shelf life at −18 °C (untreated, OD, and PEF-OD): 280, 410, and 310 days	[70]
Marine cultured gilthead seabream *^2^	2-Thiobarbituric acid reactive substances, TBARS (in mg malonaldehyde/kg muscle).	15, 10, 5, 2.5, 0, −1, and −3 °C	$\mathrm{Zero}\;\mathrm{order}\;C_{TBARs}=C_{TBARs,0}-k_{TBARs}*t$	Shelf life at −3 °C (untreated and OD): 21 and 40 days	[68]
	Pseudomonas growth (in CFU/g)		Baranyi growth model	Shelf life at −3 °C (untreated and OD): 25 and 48 days	[68]
	Total volatile basic nitrogen, TVBN (in mg N/100 g)		$C_{TVB-N}=C_{TVB-N_{0}}*\;e^{k_{TVB-N}*t}$	Shelf life at −3 °C (untreated and OD): 40 and 60 days	[68]
	Overall sensory acceptance (scoring in a 1–9 descriptive intensity scale)		$\mathrm{Zero}\;\mathrm{order}\;S=S_{0}-k_{sensory}*t$	Shelf life at −3 °C (untreated and OD): 21 and 38 days	[68]
Strawberry	Vitamin C (in mg L-ascorbic acid/100 g fresh material)	−5, −8, −12, and −16 °C	$\mathrm{First}\;\mathrm{order}\;\;C=C_{0}e^{-k_{vitC}t}$	Shelf life at −16 °C (untreated and OD with glucose, maltodextrin, and oligofructose): 74, 198, 237, and 594 days	[33]
	Color		$\mathrm{First}\;\mathrm{order}\;\frac{DC_{max}-DC}{DC_{max}-DC_{0}}=\exp\left(-k_{color}*t\right)$ (where ΔC is expressed by Equation (5))	Shelf life at −16 °C (untreated and OD with glucose, maltodextrin, and oligofructose): 216, 495, 577, and 770 days	[33]
Cucumber	Color	−5, −8, −12, and −15 °C	First order $\frac{DC_{max}-DC}{DC_{max}-DC_{0}}=\exp\left(-k_{color}*t\right)$ (where ΔC expressed by Equation (5))	Shelf life at −18 °C (untreated, OD with maltodextrin and oligofructose): 237, 402, and 331 days	[60]
Tomato	Color	−5, −8, −12, −15, and −20 °C	$DC=k_{color}*t=\sqrt{{\left(\alpha -\alpha_{0}\right)}^{2}+{\left(b-b_{0}\right)}^{2}}$	Shelf life at −18 °C (untreated, OD with maltodextrin): 133 and 161 days *	[41]
	Vitamin C (in mg L-ascorbic acid/100 g fresh material)		First order $\frac{C_{vitC}}{C_{vitC,0}}=\exp\left(-k_{vitC}*t\right)$	Shelf life at −18 °C (untreated, OD with maltodextrin): 87 and 173 days *	[41]
	Lycopene content (in mg/100 g initial content)		First order $\frac{C_{lyc}}{C_{lyc,0}}=\exp\left(-k_{lyc}*t\right)$	Shelf life at −15 °C (untreated, OD with maltodextrin): 155 and 210 days *	[41]
Watermelon	Color	−5, −8, −12, −15, and −20 °C	$DC=k_{col}*t\;DC=\sqrt{{\left(\alpha -\alpha_{0}\right)}^{2}+{\left(b-b_{0}\right)}^{2}}$		[65]
	Lycopene content (in mg/100 g initial content)		First order $\frac{C_{lyc}}{C_{lyc,0}}=\exp\left(-k_{lyc}*t\right)$	Shelf life at −12 °C (untreated, OD with oligofructose): 142 and 209 days	[65]
Green peas	Color	−3, −5, −8, −12, −16, −18, and −24 °C	First order $\frac{a}{a_{0}}=\exp\left(-k_{col}*t\right)$	Shelf life at −18 °C (untreated, OD with oligofructose, maltitol, mixture of oligofructose/trehalose): 223, 396, 325, and 357 days *	[38]
	Vitamin C (in mg L-ascorbic acid/100 g fresh material)		First order $\frac{C_{vitC}}{C_{vitC,0}}=\exp\left(-k_{vitC}*t\right)$	Shelf life at −18 °C (untreated, OD with oligofructose, maltitol, mixture of oligofructose/trehalose): 216, 347, 888, and 456 days *	[38]

*: Calculations were performed by authors, based either on digitization of published graphs or on kinetic data on E_a_ and k_ref_ parameters provided within the original manuscript. *^2^: a different secondary model was used, namely the Ratkowsky equation, $\sqrt{k}=b\left(T-T_{o}\right)$, where b is the regression coefficient and T_0_ is a hypothetical temperature, which is an intrinsic property of the organism/quality index [110].
